# Immunological challenges associated with artificial skin grafts: available solutions and stem cells in future design of synthetic skin

**DOI:** 10.1186/s13036-017-0089-9

**Published:** 2017-12-13

**Authors:** Saurabh Dixit, Dieudonné R. Baganizi, Rajnish Sahu, Ejowke Dosunmu, Atul Chaudhari, Komal Vig, Shreekumar R. Pillai, Shree R. Singh, Vida A. Dennis

**Affiliations:** 1 Center for Nanobiotechnology Research and Department of Biological Sciences, Alabama State University, 1627 Harris Way, Montgomery, AL 36104 USA; 2 Immunity, Inflammation, and Disease Laboratory, NIH/NIEHS, Durham, 27709 NC USA

**Keywords:** Skin allograft, Immune rejection, Biomaterial, Scaffolds, Stem cells, Skin tissue engineering, Skin substitutes

## Abstract

The repair or replacement of damaged skins is still an important, challenging public health problem. Immune acceptance and long-term survival of skin grafts represent the major problem to overcome in grafting given that in most situations autografts cannot be used. The emergence of artificial skin substitutes provides alternative treatment with the capacity to reduce the dependency on the increasing demand of cadaver skin grafts. Over the years, considerable research efforts have focused on strategies for skin repair or permanent skin graft transplantations. Available skin substitutes include pre- or post-transplantation treatments of donor cells, stem cell-based therapies, and skin equivalents composed of bio-engineered acellular or cellular skin substitutes. However, skin substitutes are still prone to immunological rejection, and as such, there is currently no skin substitute available to overcome this phenomenon. This review focuses on the mechanisms of skin rejection and tolerance induction and outlines in detail current available strategies and alternatives that may allow achieving full-thickness skin replacement and repair.

## Background

The skin, a subcomponent of the integumentary system, is a substantial fast-growing organ comprised of the epidermis, dermis and hypodermis layers, which in adults weighs about 7–8 pounds, covering 21–22 square feet of surface area (2-m square). The skin is a protective barrier for toxins, micro-organisms, radiation, and mechanical impacts along with regulating several physiological functions including temperature control, preventing dehydration and providing sensory detection and immune surveillance [1, 2]. Human skin frequently is damaged/injured resulting in the loss of its integrity and physiological balance, which may result in significant disability and infections. The skin’s natural restorative capacity usually is sufficient to repair and heal itself when damaged/injured [3]. However, skin grafts are required for severe skin injuries to protect the exposed layers of skin and allow the damaged portion to reform. Transplanting autologous skin grafts [4, 5] is the therapeutic approach of choice that successively reform the skin, but extensive injuries and chronic skin wounds could result in an insufficient number of autografts, especially in severe burn cases [6] and skin morbidities [7, 8]. When required, in such cases, either allogeneic or xenogeneic, skin grafts are used for transplantation. Despite allogeneic transplantations becoming more tolerant with immunosuppressive treatment, there are still some issues with early rejection. Skin allograft rejection is the recipient’s immune response following the recognition of alloantigens leading to the cellular destruction. Allogeneic or xenogeneic skin grafts may be employed but their short-term graft survival time limits their clinical use [9]. Skin allografts transplantation is employed for severe clinical cases to protect the damaged skin areas, but considering the conundrum of the rejection mechanism, the recipient may require additional transplantation from a different donor [10]. Alternative strategies are now being developed to overcome skin allograft rejections and allowing adequate skin repair [11, 12]. Novel treatment approaches include the use of stem cell-based therapies, specific immunosuppressive therapies targeting T cells or donor immune cells and skin tissue engineering. Several tissue-engineered skin substitutes are commercially available and used in clinical settings with negligible risk of immunogenic responses such as the Integra dermal regeneration template [13]. Available engineered skin substitutes are composed of either a cellular or acellular component and a biological (autologous, allogeneic, and xenogeneic) or synthetic (polymer) scaffold [14]. However, available skin alternatives engineered to mimic natural skin still do not provide a permanent solution [5, 14, 15]. This review gives an insight into different approaches and innovative advances to allow overcoming skin allografts rejection.

## Immunological rejection

### Mechanisms of skin graft rejection

Allografts have been used for many years in transplantation; however, donor tissue availability remains a critical issue. Cadaver tissues, especially organs, are in high demand and harvesting of skin has to be completed rapidly [16] post-death and preserved [17]. Critical issues associated with allografts are availability and rejection. Laboratory-grown artificial tissues are now in development to help overcome the immunological rejection issues [18, 19]. Over the years, synthetic skins comprised mostly of human cell lines with biodegradable materials have been used for transplantation onto burned and wounded skin patients [20, 21]. Even though the artificial skin products are in development and available commercially, they are still prone to rejections [7].

Skin autografts transplantation is a well-known medical procedure. Grafting between genetically identical individuals (syngeneic graft) can be successful without a prolonged immunosuppressive treatment. Even though immunosuppressive treatments for organ transplantation are effective in preventing early rejection, skin tissues whether from both donor or engineered are continuously failing [22]. Skin graft successfully placed at the donor site but rejected within 1–2 weeks is consistent and is termed first set rejection. The second set of rejection is even faster if grafted from the same donor. Graft rejection is an intricate mechanism, which involves an array of processes and ultimately potent inflammatory responses initiated by innate immune responses and destruction of the donor tissue [23]. The rate of rejection of donor tissue at the recipient’s graft site is dependent on the graft volume and antigens incompatibilities between both. The role of T lymphocytes in graft rejection is vital as evidenced from studies in nude mice, which do not reject allogeneic skin grafts because they lack CD4+ and CD8+ functionality [24]; however restoring this functionality with adoptive transfer of normal T cells initiates rejection of the skin graft [25, 26]. Therefore, a hurdle in allogeneic skin grafting is the triggering of CD4+ and CD8+ T cells immune responses, sometimes involving both for first set rejection, although second set rejection could be facilitated by antibodies [26].

**Fig. 1:**
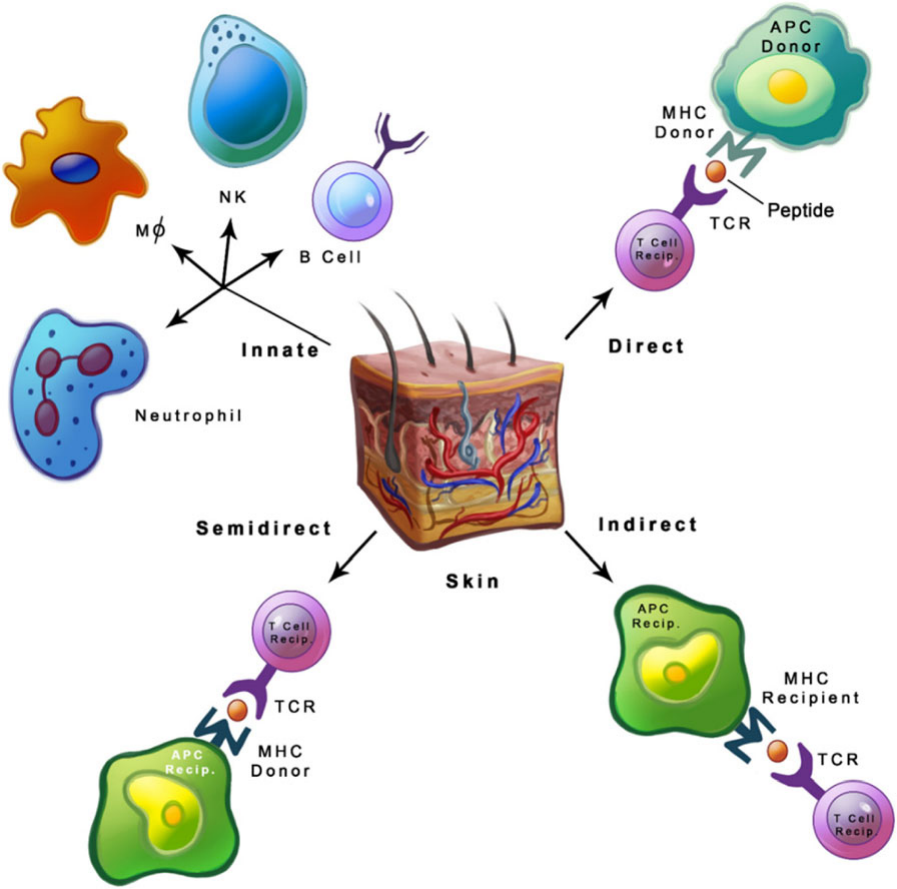
Allorecognition pathways: Direct pathway is the process whereby the donor’s MHC molecules on APCs is recognized by the TCRs of recipient’s T cells. Indirect pathway recognizes the processed peptide presented by recipient’s MHC on APCs. Semi-direct pathway is where T cell activation occurs by transfer of the donor’s MHC onto the recipient’s APCs

The mechanism of skin graft rejection (Fig. 1) starts with responses from dendritic cells (DCs), macrophages, polymorphonuclear cells, angiogenic mediators, and cytokines to promote rejection [22, 23], followed subsequently by the activation of T cells (CD4+ and CD8+). Further, accumulation of inflammatory cytokines and effector T cells permeate the skin graft to commence rejection [22, 26, 27]. The event/stimulus that triggers skin graft rejection arises from a mismatch in donor MHC and recipient T cells receptors (TCRs) [28]. Even though matching the MHC type is critical in avoiding skin grafts rejection, a single genetic difference at the loci of MHC molecules can still commence the rejection process by stimulating alloreactive T cells [10]. Additionally, even if the rejection rate is not very high in genetically related donor and recipient [29], it can be controlled by immunosuppressive drugs. The only scenario where allograft transplantation without immunosuppressive drugs is successful when the donor and recipient are identical twins, with true human leukocyte antigen (HLA) match [30], which shows the immunological importance of MHC molecules in rejection of transplants. Thus matching the HLA types [31] between non-identical twins improves the rate of graft transplantation, but HLA typing methods are not precise due to complexity and polymorphism of MHC molecules [32]. Another reason is differences in minor histocompatibility antigens (MHA) that also vary in individuals for HLA type matching, which is a consideration in assessing graft rejection [33].

Most allografts require MHC class matching for allogeneic transplantation, and the primary reason for a rejection response against foreign MHC molecules is TCRs specificity. Foreign MHC antigen recognition between both the recipient and donor tissues initiates the rejection process at the graft site. The repercussion of allorejection is the initiation of adaptive immune responses especially with alloreactive T cells [22]. The allorecognition mechanism for skin grafts is distinguishable from other tissue transplantations. Apparently, skin graft rejection is potentially a much broader response generated to destroy the donor skin graft. The demonstration on corneal transplant rejection in mice indicates that only the CD4+ indirect pathway involving minor antigens leads to rejection. The CD8+ T cells pathway has limited functionality but no cytotoxicity [34].

Dendritic cells from donor grafts also have a crucial function in initiating the rejection process by their migration [35] into the donor’s lymphoid organs for antigen presentation to initiate the adaptive immune response [36]. The adaptive alloimmune response from DCs is attributed to Langerhans cells (LCs) from the epidermis and dermis. Langerhans cells are distributed in the epidermis and contribute up to 3% in epidermal cells [22]; they express CD1a in humans and have the capability of microbial lipid antigen presentation to T cells [37].

### Adaptive immunity in allorejection

#### Direct allorecognition

Direct allorecognition pathway (Fig. 1) is the outcome of the interaction between the recipient’s T cells in the lymphatic system with foreign MHC molecule from migrating leukocytes of the donor. Direct allorecognition exploits CD4+ and CD8+ subsets of T cells for identification of MHC class I and II molecules from a donor, but antigen processing by the recipient’s antigen presenting cells (APCs) is not mandatory. This rapid donor-recipient recognition generates robust T cells responses [22] with alloreactive activated T cells from the recipient migrating to the donor tissue for the direct attack [10]. The donor MHC class II and class I molecules recognition by the recipient’s CD4+ and CD8+ T cells respectively generate a robust response. Although recognizing foreign MHC molecules is not precisely the nature of T cells, the observed cross-reactivity between self and foreign MHC molecule suggests that some non-specific T cells might be involved in this process [22, 28]. This phenomenon of foreign MHC molecules recognition was proposed with TCRs similarities on donor’s and recipient’s T cells to identify the nominal and allogeneic MHC proteins [38] and cross-reactivity [26, 30]. The lack of specificity in the direct pathway proposes the plausible cause for transplant rejection even in highly matched MHC molecules between donor and recipient [30] and corroborate that even a single variation in MHC molecule can promote graft rejection [10, 22]. Leukocytes depletion in donor tissue prolonged allograft survival but remained ineffective in averting the rejection process [10]. In a study using mixed lymphocytes in vitro, where allorecognition points towards the non-specificity of antigen recognition, and in vivo with mice lacking CD4+ T cells, where the indirect pathway triggering via antigen presentation is not possible, CD8+ T cells alone were observed to be functional in graft rejection [39–41].

Dendritic cells of donor grafts are the key initiators for direct pathway activation in the graft recipient’s immune system. This premise was proven by depletion of the donor’s DCs which halted the immunogenicity, as the later addition of the donor’s DCs reverted the whole process [42]. Diminishing donor APCs via antibody treatment [10] may propose an alternative mechanism in avoiding the direct allorecognition process to prevent rejection. Similarly, alloreactive T cells activation via endothelial cells may pose an alternate process in the allograft recognition [26]. The robust T cell response generated in the direct pathway by itself can cause complete rejection, but alloantibodies production is not the outcome of direct allorecognition pathway [43].

#### Indirect allorecognition

Allorecognition via the indirect pathway (Fig. 1) is primarily contributed by the recipient’s APCs presenting foreign proteins to T cells from grafts and activating CD4+ T cells. The indirect pathway is based solely on CD4+ T cells. The robustness of indirect allorecognition is significantly less than direct allorecognition where CD4+ and CD8+ T-cell phenotypes partake without antigen processing but are adequate for rejection of grafts. The indirect pathway is evidence of the conventional antigen processing and presentation by APCs. Even though the indirect pathway represents specificity, the rejection is also very often due to minor H antigen loci differences [10, 33].

The specificity of the indirect pathway was demonstrated in a rat model by priming the indirect allorecognition and evading the direct allorecognition [44] to confirm this pathway’s involvement in graft rejection. In this experiment, the majority of CD8+ T cells were depleted in mice via injecting an anti-CD8 monoclonal antibody that resulted in a dominant Th2 response. However, the overall contributory role of indirect allorecognition in the immunological graft rejection could not be validated when tested alone [45]. Although the indirect pathway is the basis for long-term rejection, it can also activate macrophages, thus resulting in tissue injury and fibrosis, furthermore developing alloantibodies against allografts [46].

The indirect pathway selectively depends on CD4+ T cells, but the support of CD8+ T cells is also evident; a process termed cross-dressing [22]. CD8+ T cells participation in graft rejection was underscored by in vivo experiments with single MHC class I peptide presentation followed by confirmation of skin allografts rejection [47, 48]. Allorecognition by CD8+ T cells also initiates the acute allograft rejection, but activation of the CD8+ T cells dependent indirect pathway may require support from CD4+ T cells [41, 49]. Evidently, the indirect pathway remains the assertive mechanism in long-term allorecognition as long as the allograft remains on the recipient’s graft site and role of memory T cells remains affirmative in rejection and tolerance [26, 48].

#### Semi-direct allorecognition

Direct allorecognition is the most dependable pathway in rejection followed by the indirect pathway, but the alternate pathway known as cross-dressing has been reported [22, 50]. In this semi-direct allorecognition pathway (Fig. 1), the recipient APCs acquires intact MHC molecule from the donor for antigen presentation to T cells; this contrasts to the indirect pathway where the processed peptides of allogeneic MHC molecules on the recipient’s MHC molecules are displayed by the recipient’s APCs [22]. This process indicates that the same APCs present MHC class II and I molecules for CD4+ and CD8+ T cells activation [23]. This controversial representation of both MHC molecules, which are presented by the same DCs and referred to as “three-cell interaction” [51] was supported by staining the MHC molecules and inducing T-cell specific proliferative responses [52]. This sharing of MHC molecules has subsequently been explored using different DCs subsets and was observed to be a natural phenomenon of DCs for transfer efficiency [53]. Such recognized DCs presenting MHC class I and II molecules was further documented in mice [52]; however, the semi-direct pathway involvement in graft allorecognition was not evident in in vivo studies [50]. The mechanism of the MHC transfer apparently is via exosomes [54, 55] that are released by multiple cell types and hypothesized to be representing the MHC molecules onto their membranes. The interaction of MHC class I molecule-deficient DCs, and CD8+ T cells specific to an antigen with exosomes accommodating MHC molecule revealed the transfer of molecules from exosomes to DCs [54]. Substantiation of allogeneic MHC molecule transfer undoubtedly indicates the semi-direct pathway involvement, but this pathway’s precise role in allograft rejection still warrants further investigations.

#### B cells

Allograft recognition is dominated mainly by T cells although B cells are thought to be involved. Pre-existing alloantibodies against blood groups and polymorphic MHC antigens are reasons for antibody-mediated allograft rejection, and B cells also have the capability of antigen presentation [23]. Both antibody production and the antigen presentation capability of B cells potentially participate in allograft rejection [56]. Others have proposed that B cells can promote T cells activation via co-stimulatory pathways and cytokines release [26, 57]. Alloreactive memory T cells also represent the diverse functionality of T cells in allograft rejection [58]. Moreover, recent investigation for comprehending the diverse functionality of B cells in allograft rejection revealed that proliferation, differentiation, and memory T cells functionality were increased, which may be due to B cells ability to function as APCs [58]. B-cell deficiency in mice further suggested that the extended graft survival rate resulted from the absence of antigen presentation functionality [59]. Depleting mature B cells with anti-CD20/anti-CD19 monoclonal antibodies in mice accelerated the rejection of skin allografts [60, 61] by enhancing the allospecific memory T cell immunity, which may explain B cell participation in allorecognition and survival [56–61].

### Innate immunity in allorejection

Components of innate immunity that participate in allorejection include natural killer (NK) cells, macrophages, monocytes, and neutrophils (Fig. 1). Natural killer cells specialize in killing target and apparently participate in indirect allorecognition of the allograft by activating CD4+ T cells [62]. Depletion of NK cells improved the rate of cardiac allograft [63] acceptance and extended the survival of corneal allografts [64], while functional NK cells were found to assist the CD4 mediated allograft rejection [65]. However, responses elicited by NK cells alone are insufficient for skin allograft rejection [66, 67]. Results from an in vivo study using T and B cells deficient, but IL-15 producing NK cells in mice revealed acute skin allograft rejections [68]. IL-15 is required for memory T cells survival and development, but not induction of memory phenotypes [67]. Natural killer cells reportedly participate in both tolerance [69] and rejection [70] of allografts. The participatory role of NK cells in allograft tolerance is further substantiated by their destruction of donor APCs [68], secretion of IL-10 [69] and arrest of CD8+ memory T cells proliferation [71]. Macrophages do not play a direct role in allorejection since they are not efficient in priming naïve T cells [72, 73]. Macrophages are heterogeneous depending on their functions and are in large numbers in human allotransplants. In general, macrophage activation follows the classically activated M1 and alternatively activated M2 phenotypes. M1 macrophages are activated by Th1 secreted cytokines, i.e., IFN-γ and TNF-α [22, 73, 74]. M2 macrophages are induced by IL-4 produced by Th2-activated T cells as well as basophils and mast cells in response to injuries [75]. Macrophages are frequently observed in acute rejections, which may indicate their defensive functions in necrosis and pro-inflammatory cytokines secretion.

Neutrophils participate in allograft rejection by secreting chemokines thus leading to T cells activation and proliferative responses [71]. The role of innate immunity elements in immunological rejection of allografts is not highly evident. However, support of adaptive immune responses via innate immunity cells may be a more logical explanation since they are rapid responders against foreign molecules. Overall, the process of allograft rejection is by the direct, indirect and semi-direct pathways, but innate immunity components may participate along with adaptive immune responses to boost the allograft rejection process.

## Approaches to avoid skin immune rejection

### Therapies to escape skin rejection

The use of skin autographs is the most efficient method and the treatment of choice to avoid immunogenicity in reconstructive skin transplantation. However, there are limitations in using skin autografts for patients with deep and/or large wounds or with extensive burns [76, 77]. In such circumstances, transplantation of split-thickness skin allotransplants or full-thickness skin from live donors or in the form of cadaveric skin provides a replacement that reforms the functional skin [22, 76]. Nonetheless, there are limitations to the use of allogeneic skin grafts since invariably they are rejected due to the triggering of the host immune response that subsequently leads to their short-life span [22, 76, 78]. Furthermore, efficacious immunosuppressive treatments usually used in organ transplants to prevent early rejection are either less or/not effective in skin transplantation [77]. Treatment of skin allografts before operation allows decreasing the immunogenicity, but it is insufficient over the long-term [78]. Newer treatment procedures have thus been developed to overcome skin grafts rejection to prolong skin graft survival.

#### Therapies addressing donor-derived DCs

A large number of researchers have reported on effective therapies addressing donor-derived DCs to induce skin graft tolerance. Indeed, as donor-derived DCs are critical in acute immune responses in skin transplants underlying allograft rejection, there is every likelihood that their depletion or inhibition results in prolonged survival of skin grafts [78, 79]. Most DC-based strategies aimed at inhibiting the antigen presentation process predominantly by targeting donor-derived LCs and the DC-subpopulations expressing MHC class II glycoproteins are important in the initiation of allograft rejection [22, 78]. Strategies using chemical agents to modulate the activity of DCs have shown a high improvement of skin grafts survival. For example, treatment of skin grafts with Gliotoxin, an epipolythio dioxopiperazine (ETP) immunosuppressive mycotoxin, significantly reduced the epidermal density of LCs and altered their function, resulting in the enhancement of skin graft survival and induction of donor-specific tolerance onto MHC-mismatched recipient mice [80]. Gliotoxin apparently immunomodulates the functions of immunocompetent cells and reduces contact hypersensitivity responses through the induction of suppressor cells thus leading to inhibition of graft rejection for prolonged survival without altering the skin functions [80]. Treatment of skin grafts with 10-dimethyl-1,2-benzanthracene (DMBA) depleted LCs and therefore the class II MHC antigens from the graft, which enhanced the survival of C57BL/6 skin grafted onto BALB/c recipients [81]. Norcantharidin (NCTD, C_8_H_8_O_4_), a cantharidin that promotes hematopoiesis extended the survival time of allogeneic skin grafts in mice by modulating the activity of DCs function towards tolerance and inducing immune silencing via inhibiting the activity of calcineurin phosphatase [82].

Despite the above examples, approaches employing chemical agents are limited by the potential risk of toxicity that restricts their clinical use. Therefore, alternative therapeutic methods that do not have, or exhibit limited, adverse effects have been developed. An example is photodynamic therapy (PDT) employing a light-sensitive drug, aminolevulinic acid (ALA) and methyl aminolevulinate (MAL) as a photosensitizing agent and a non-thermal light to activate the drug [83] increased the persistence of skin allografts in mice pre-treated with PDT [83, 84]. PDT also down-regulated both MHC molecules and B7 expression levels on donor skin-derived epidermal LCs and rendered LCs unable to activate allogeneic T cells proliferation, consequently leading to prolongation of fully histo-incompatible skin allograft survival [84]. Skins of C57BL/6 mice pre-treated with verteporfin and light (λ = 690 ± 10 nm) remarkably extended the survival of skin allografts on recipient BALB/c mice [84]. Human skin allografts treated with an antibody against β2-microglobulin (β2mAb) and ultraviolet-C light (UVC) irradiation prolonged skin survival compared to the untreated skin in severely burned patients [85]. The long-term skin survival effect of this treatment resulted from β2mAb, which impaired the functions of HLA-class I antigen, and UVC-treatment which reduced the number of skin APCs for an efficiently localized immunosuppression [85]. UVC-treatment inhibits the induction of contact hypersensitivity responses by depleting LCs and limiting their migratory capacity [86–88]. Furthermore, it induces the release of epidermal growth factors, promotes proliferation of endothelial cells, restores melanin production which accelerates wound healing and restoration of skin homeostasis [89, 90]. Also, UVC-treatment of dermal fibroblasts increases the release of fibronectin in the cellular microenvironment for the contraction of fibroblast-populated collagen lattices, thereby resulting in increased healing through wound contraction [90, 91].The loading of donor’s antigens with donor-derived immature DCs and third-party DCs was also reported to partly induce skin transplantation tolerance against rejection in mice [92].

#### Inactivation and deletion of alloresponsive T cells

Approaches exploiting the inactivation of functional T cells subpopulations have been revealed to induce durable tolerance and allograft survival. Activation of T cells by recognition of allogeneic skin grafts is sufficient to initiate acute rejection. Supposedly, this is accomplished by inducing CD4+ and CD8+ phenotypic activation with subsequent production of the Th1 pro-inflammatory cytokines, IL-2 and IFN-γ [22]. In mice, the endogenous ligand for FMS-like tyrosine kinase 3 (Flt3 ligand, FL) stimulated robust tolerance of skin grafts in recipients of FL-mobilized donor cells [79]. FL-induced skin graft tolerance was inherent to the durable macro-chimerism of persistent blood and selective suppression of donor-reactive T cells [79]. Furthermore, treatments based on antibodies with immunosuppressive effects induced tolerance ultimately leading to the survival of skin grafts. Use of the FN18 antibody specific for the CD3 antigen of rhesus monkey T cells significantly extended the survival of skin grafts in rhesus by modulating or depleting T cell subsets [93]. Anti-Ly49A mAb YE1/48 reactive against the Ly49 receptors expressed on T cells, NK, and NKT, regulate immune responses through inhibition/activation of MHC class I molecules were shown to delay MHC molecules-mismatched allogeneic skin graft rejection in mice [94]. YE1/48 mAb prolonged the survival of skin grafts by inhibiting only the primary immune responses to allografts [94].

Treatment with an anti-CD80 mAb combined with cyclosporin A, an immunosuppressive drug, suppressed the activation of T cells and triggered alloantigen-specific non-responsiveness resulting in significant increase of skin grafts survival in a preclinical rhesus monkey model [95]. Transfusion of C57BL/6 mice recipients with donor BALB/c spleen cells and anti-CD154 antibody also permitted skin grafts acceptance and survival [96, 97]. Prolonged rat skin xenografts survival occurred following transfusion of mice with donor-specific cells and monoclonal an anti-CD154 mAb [97]. The longevity of graft survival provided by this treatment entailed the continuous activation of CD4+ and alloresponsive T cells without IFN-γ in the graft [96]. Furthermore, prolonged allografts survival times were observed in recipients treated with donor-specific transfusion and an anti-CD154 mAb essentially by deletion of alloantigen-specific CD8+ T cells, which led to an allotolerant state [98–100]. In addition to CD8+ T cell deletion, the initiation of skin allograft survival required CD4+ T cells, but other mechanisms along with different CD4+ T cells subsets can induce skin transplantation tolerance [100].

The success of T cells depletion approaches relies on the generation of stably mixed chimaerism in which host T cells are ablated to achieve tolerance of donor’s MHC mismatched grafts [101]. The clinical application of this approach, however, has been limited by the need for pre-transplant treatment with myeloablative agents, their potential toxicities and split tolerance due to unmatched minor antigens [101–103]. Also, post-depletion of humoral responses and the repopulation of memory T cells without xenogeneic antibody production and/or over-immunosuppression represent a considerable challenge [103]. Even though T cells depletion approaches proved highly effective in animal models, the matching of minor antigens to prevent effector T cells from rejecting donor skin grafts might not be possible in clinical practices due to the lack of effective in vivo T cell depleting agents [101–103].

#### Bioengineering

Bioengineering appears to be a promising alternative therapy for long-term skin graft acceptance and survival. Bioengineered acellular matrices have a high potential to improve healing outcomes and survival rates while reducing immunogenic and/or secondary complications [78, 104, 105]. Typically, acellular dermal matrix membranes are composed of collagens, fibronectin, glycoproteins, lamellar, integrin, and synthetic biopolymers [78, 104, 105]. A bioengineered acellular membrane made of a complex pattern of collagen type IV, proteoglycans and glycoproteins applied between the wound surface and skin allograft substantially delayed the onset of acute skin allogeneic graft rejection in mice [76]. This artificial interface interrupted the normal effector pathway which resulted in prolonged skin allograft survival without immunosuppression [76]. Employing the nano-barrier, NB-LVF4A membrane to skin allografts and wounds similarly extended the survival of skin allograft without triggering immunosuppression [106]. The bioengineered interface of the acellular matrix membrane provides a physical barrier between the recipient and donor tissues for interrupting the effector pathway to protect from the allorecognition pathway underlying the humoral rejection [76]. The network of adhesive molecules of bioengineered membranes acts as a barrier to cellular migration while, at the same time, it grants free diffusion of nutrients and oxygen [76]. It is still not well understood how bioengineered membranes provide protection that results in prolongation of allograft survival especially when mature complex capillaries, arterioles, and venules have developed [76].

#### Gene therapy

Gene therapy is also a promising approach to induce tolerance and effectively extending the survival time of skin allografts. Transduction of hematopoietic fetal liver cells with human IL-10 (hIL-10) gene before transplantation delayed rejection and lengthened the survival time of mouse skin allografts [107, 108]. Overexpression of IL-10 was achieved by inserting the IL-10 plasmid into GPE86 fibroblastic cell line to produce retroviral vectors carrying the hlL-I0 gene [107].This provision by IL-10 is because it is an immune-regulatory cytokine that exerts its immunosuppressive activities by inhibiting the synthesis of Th1 cytokines [107, 108]. Therefore, regulating the effects of T cell responses through overexpression of IL-10 in the donor can induce long-term tolerance and improve graft survival. Donor hematopoietic stem cells transduced with hIL-10 extended the survival of donor skin allografts through the continued production of IL-10 and induction of donor cell chimerism and engraftment which protected allogeneic grafts from rejection [107, 108]. However, a full tolerance was not attainable with this treatment strategy. Transgenic expression of human CTLA4Ig (cytotoxic T-lymphocyte-associated antigen 4-immunoglobulin) reportedly also lengthen the survival of xenogeneic skin grafts on burn wounds in rats and mice [109, 110]. Cytotoxic T-lymphocyte-associated antigen 4-immunoglobulin modulates T cells functions by competitively inhibiting the CD28 and B7 co-stimulatory pathways [109, 111]. Hence, CTLA4Ig by down-regulating activated T cells could induce transplantation tolerance and reduce immune rejections. Transgenic CTLA4Ig locally inhibited human lymphocyte activation and proliferation without significantly affecting the systemic immune function which led to prolonging grafts survival of the transgenic skin [109, 110]. Furthermore, transfection of allogeneic skin flaps with CTLA4Ig and OX40Ig gene mediated by lentivirus vectors significantly increased the survival time of tissue allografts in rats [112]. OX40 is a subcomponent of the TNF superfamily of receptors involved in T cell co-stimulation [78, 112]. Local transfer of OX40Ig and CTLA4Ig genes inhibited the rejection of allografts and expanded survival time by decreasing the quantity of CD4+ T cells, increasing the clonal expansion of T helper subset 2 (Th2) subpopulations and down-regulating IL-2 and IFN-γ expressions [112]. Skin gene therapy holds great promise in allotransplantation tolerance and improvement of long-term allografts survival. Gene therapy provides the advantage of a local production of immunosuppressive molecules. Cells or organs are treated ex vivo with gene transfer vectors before implantation allowing the production of immunomodulatory proteins in the donor grafts and resulting in local rather than systemic immunosuppression [113]. However, some key risks to consider are the fact that the host immune response limits repeated administrations of the vector and safer vectors need to be developed [114, 115]. Moreover, although the gene expression and protein production are transient, the introduced mutagenesis, the immunogenicity and alloimmune response, and vector stability in the host represent important clinical challenges to avoid endangering patients [115, 116]. Challenges also include the development of more efficient and durable vectors for sustained expression of the desired gene in vivo with minimal toxicity principally in regards to genomic integration and immune response [113, 115–117]. Noteworthy of mention is the difficulty in achieving a specific and uniform therapeutic transfer to different compartments of the skin that must be addressed [117].

#### Antioxidant therapy

Antioxidant therapies of donor skins from C57BL/6 mice before transplantation, or BALB/c mice recipient skins with Salen-Manganese (Salen-Mn) complexes were demonstrated to delay allograft rejection [118]. Salen-Mn delayed allograft rejection and increased skin allograft survival by reducing reactive oxygen species (ROS)-mediated graft tissue damage, by reduction of anti-donor cytotoxic responses via the decrease of Th1 alloreactive cells and increase of donor-specific Th2 cells, and by suppression of inflammatory reactions [119]. However, the precise mechanism underlying the promotion of anti-inflammatory T cell responses was not elucidated in these studies.

#### Skin tissue engineering to overcome rejection

**Fig. 2:**
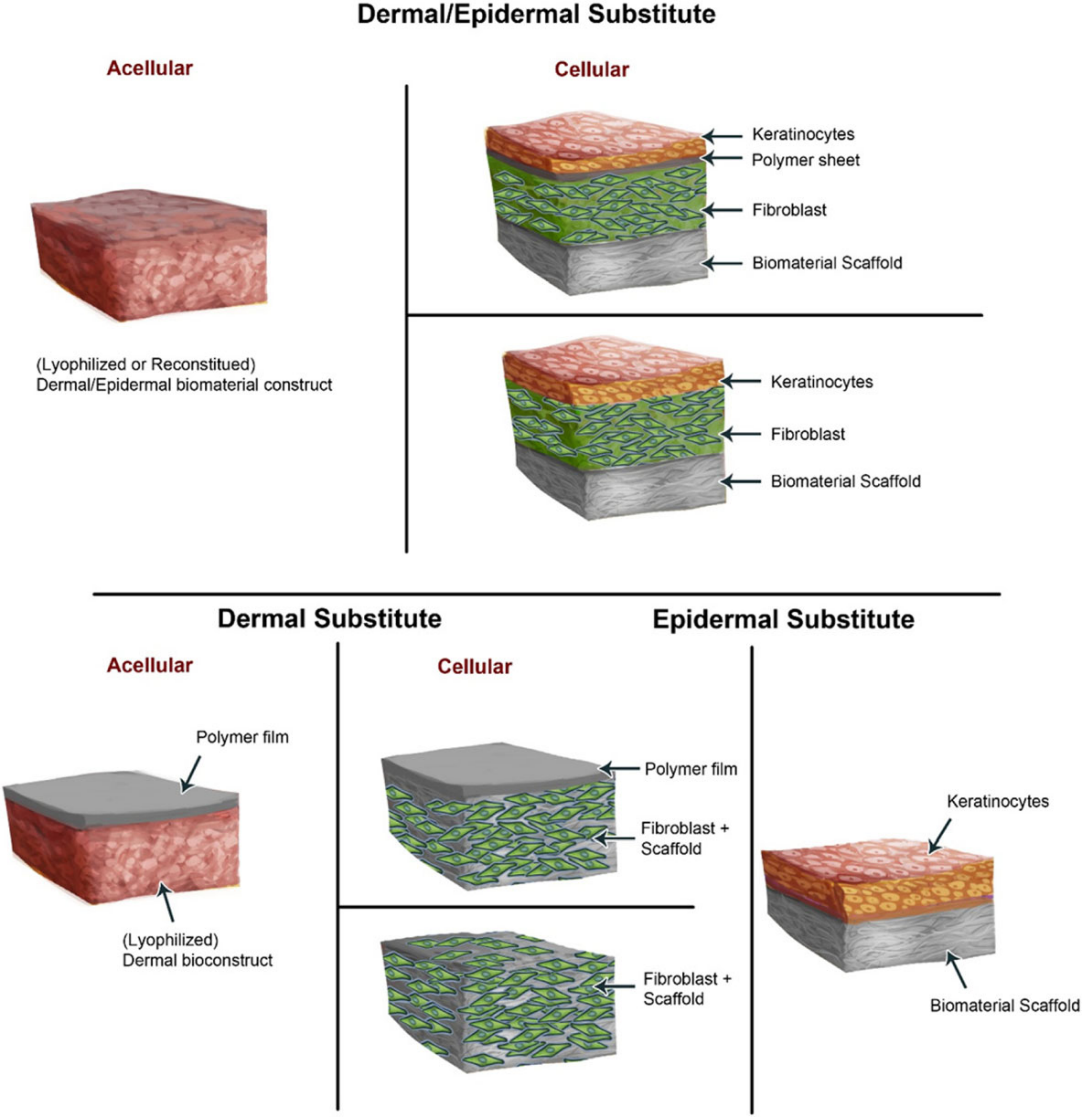
Bioengineered skin substitutes models. Tissue-engineered skin bio-constructs are either acellular or cellular and are composed of epidermal and/or dermal equivalents most often enclosed into a matrix scaffold of biomaterials

In skin tissue regeneration, repair and transplantation, especially for extensive skin injuries, biomaterials that support skin cells for implantation offer an alternative approach to promote healing and obtain long-term and complete restoration of damaged skins. The development of bioengineered skins has led to the emergence of artificial skins incorporating an extracellular matrix of biomaterials and cells (autologous cells, allogeneic cells or xenographic tissues) with minimum risks of rejection (Fig. 2) [12, 120–122]. Bioengineered skin substitutes act first as protective dressings to limit infection and fluid loss and further function as skin equivalents to provide temporary wound covers or permanent skin replacements [123, 124]. Bioengineered skins are either acellular or cellular and are composed of epidermal and/or dermal equivalents enclosed into a matrix scaffold of biomaterials which are further incorporated into the body during the regeneration of new skin (Fig. 2) [121, 125, 126].

Biomaterial components used to make skin substitutes include natural and synthetic materials that provide a matrix scaffold onto which grafted skin cells grow and spread [121, 124]. Some natural materials include fibronectin, collagen, chitosan, hyaluronan, and glycosaminoglycans (GAGs) [121]; synthetic materials include synthetic polymers like polyethyleneglycol (PEG), poly lactic-co-glycolic acid (PLGA) and natural polymers like dextran, chitosan, gelatin, fibrin, and hyaluronic acid [127–130]. Biomaterial scaffolds are designed in a solid and porous three-dimensional (3D) form with the aim of performing several functions including promoting interactions between cells and the biomaterials and the deposition of the extracellular matrix (ECM).). Due to cell size along with cell migration requirements and transport, the optimal scaffold pore size is usually around 100 μm, but pore sizes greater than 300 μm are highly recommended to achieve, especially, the formation of capillaries [131]. This porous scaffold permits sufficient transport of nutrients, regulatory and growth factors for proliferation, differentiation, and survival of cells. The scaffold also undergoes controlled biodegradation while supporting tissue regeneration with similar physical, mechanical and functional properties; and inducing a minimal degree of toxicity, immunogenicity and inflammation [124, 132]. These biomaterials particularly enable to overcome limitations of rapid and permanent implementation of the grafted skin while reducing the incidences of infection and rejection [121, 122]. Nevertheless, there are no bioengineered skin substitutes to completely replicate skin or fulfill all its functions mentioned above [12, 121].

### Immune response to bioengineered skins

The implantation of bioengineered skins elicits a series of host immune reactions, first towards the cellular component and further to the biomaterial component [133]. However, there is lack of reported studies regarding the host immune responses to biologic scaffold materials, and those available studies mostly evaluate the inflammation response. The biomaterial implantation initiates inflammation responses through a series of events, collectively known as foreign body response starting with proteins adsorption from the ECM on the biomaterial surface followed by the attraction, adhesion, and activation of phagocytes such as monocytes and/or macrophages in the implant site [133–135]. Phagocytic cells might also be attracted to the inflammatory site by molecular patterns that mimic pathogen-associated molecular patterns (PAMPs) that may be on the biomaterial, through innate receptors or the recognition of proteins adsorbed to the biomaterial by APCs [134]. Activated macrophages then secrete a wide range of cytokines (i.e., IL-1, IL-6, IL-10, TGF-β), chemokines (IL-8, MCP-1, and MIP1-α/β), growth factors and ECM enzymes [135, 136]. Depending on the biomaterial and cellular component in the bioengineered skin, these mediators could direct the skin tissue repair and inflammatory response to the biomaterial or mediate other variable responses including the migration and proliferation of fibroblasts and skin tissue regeneration [133, 135, 137].

Moreover, surface contact of complement proteins with biomaterials and the adsorbed protein layer triggers the complement cascade that leads to the activation of inflammatory cells, which subsequently mediate other processes, including maintenance of inflammation, activation, and initiation of tissue repair or promotion of T and B cells development [133, 136]. Hence, selection of biomaterials for bioengineering of artificial skin tissues depends, in addition to addressing functional skin requirements, on the potential host responses towards them. However, although biomaterials can cause inflammation, they contribute minimally to transplant rejection [133, 135]. Furthermore, the elimination or inactivation of cellular elements that lead to immunogenic responses from the matrix scaffold (i.e., cells and cellular antigens) allows the artificial skin to exhibit minimum early rejection and not to cause a chronic rejection reaction after implantation [105, 138].

### Skin substitutes with natural biomaterials

Naturally occurring biomaterials capable of reproducing the micro-architecture and physiological functionality of the ECM are more widely used in designing skin substitutes, and they include fibronectin, collagen, chitosan, hyaluronan, and glycosaminoglycans (GAGs) [121]. Their main advantages reside in their inherent properties of biological recognition, low antigenicity, biodegradability, low toxicity and low chronic inflammatory responses [125, 139–141].

#### Collagen

Collagen-based matrix scaffolds are the most attractive matrix for artificial ECMs. Collagen, an essential structural component of the ECM, comprises more than 70% of the dry weight of the dermis [142, 143]. Over twenty different types of collagens exist of which Type I and III are more abundant in dermal tissues, while Type IV and VII are the major components of the basement membrane [121, 124, 142–145]. The advantage of using collagen resides in its high biocompatibility, biodegradability, and weak antigenicity while offering support for cell attachment and growth compared with other natural biomaterials [141, 142]. The use of collagen, especially of animal origin is currently widespread for the development of in vitro three dimensions (3D) full thickness skin equivalent models that exhibit close morphology and metabolic activity of human skin to study skin biology, wound healing, and skin cancer [146, 147]. The improvement of culture techniques has led to a successful commercialization of artificial human skins based on collagen as their biomaterial component (Table 1).

**Table 1:** Some current commercially available bio-engineered skin substitutes

Type	Brand	Components				Indication	Permanent Cover	References
		Scaffold Material	Scaffold Source	Cell Component	Cell Source			
Epidermal Substitutes	Bioseed^®^	Fibrin sealant	Allogeneic	Keratinocytes (cultured)	Autologous	Wound treatment	Yes	[158, 159]
	Laserskin^®^	Benzyl-esterified Hyaluronan derivative	Recombinant	Keratinocytes / Fibroblasts (cultured)	Autologous/ Allogeneic	Regeneration and skin resurfacing for Burn wounds & Chronic full thickness ulcers	Yes	[161, 165, 167]
	MySkin™	Silicone layer	Synthetic	Keratinocytes (cultured)	Autologous	Neuropathic, pressure & Diabetic foot ulcers	Yes	[11, 184]
Dermal/Epidermal Substitutes	Apligraf^®^	Type I Collagen	Bovine	Keratinocytes and fibroblasts (cultured)	Allogeneic	Partial & Full thickness burns, chronic wounds, Leg & Foot ulcers	No	[6, 11, 101, 120]
	OrCel™	Type I Collagen sponge	Bovine	Keratinocytes and fibroblasts (cultured)	Allogeneic	Healing of autograft donor sites, Reconstruction of recessive dystrophic epidermolysis bullosa	No	[6, 11, 101]
	EZ Derm^®^	Aldehyde-cross linked Collagen	Porcine			Dressing for partial thickness burns	No	[11, 120, 145]
	PolyActive	Polyethylene oxide terephthalate & Polybutylene terephthalate (PEO/PBT)	Synthetic	Keratinocytes and fibroblasts (cultured)	Autologous	Dressing for partial thickness wounds	No	[11, 183]
	GammaGraft™	Cryopreserved Collagen	Cadaveric & Allogeneic			Skin graft for burns, chronic wounds	No	[100]
	MyDerm™	Fibrin	Autologous	Keratinocytes and fibroblasts (cultured)	Autologous	Coverage of full thickness skin loss	Yes	[116] [152, 157]
Dermal Substitutes	Integra®	Type I Collagen & Chondroitin-6- Sulfate	Bovine			Partial and full thickness wound, burns, Chronic ulcers	No (Semi)	[6, 11, 101, 120, 121]
	PriMatrix^®^	Type I and III Collagen	Bovine			Coverage of complex wounds	Yes	[99, 126, 127]
	Alloderm^®^	Lyophilized Collagen	Cadaveric & Allogeneic			Resurfacing of full thickness burn wounds & Wound cover	Yes	[100, 101, 120]
	Oasis^®^	Type I, III and V Collagen	Porcine			Wound covering	Yes	[6, 99, 120]
	GraftJacket^®^	Cryopreserved Collagen	Cadaveric & Allogeneic			Various wound repair	Yes	[100]
	Permacol^®^	Collagen and Elastin	Porcine			Skin dressing	Yes	[99, 121]
	MatriDerm^®^	Type I Collagen & Elastin	Bovine			Regeneration of full thickness burn & chronic wounds	Yes	[6, 99]
	Trancyte^®^	Type I Collagen, Nylon mesh, Silicon film	Porcine & Synthetic	Neonatal fibroblasts (cultured)	Allogeneic	Wound dressing for partial thickness burns	Yes	[11, 121, 144]
	Biobrane^®^	Type I Collagen, Nylon filament, Silicon film	Porcine & Synthetic			Regeneration & Wound dressing for Partial & Full thickness wounds and Chronic ulcers	Yes	[6, 144, 230]
	Dermagraft^®^	Polyglycolic acid/Poly(lactic acid) (PGA/PLA) & ECM	Synthetic & Allogeneic	Neonatal fibroblasts (cultured)	Allogeneic	Covering for Burns & Chronic wounds & Chronic diabetic foot ulcers	Yes	[6, 11, 127, 182]
	Hyalograft 3D	Hyaluronan	Allogeneic	Fibroblasts (cultured)	Autologous	Deep burns & Foot ulcer treatment	Yes	[11, 169]
	Hyalomatrix^®^	Hyaluronan (HYAFF) on silicone layer	Allogeneic & Synthetic			Wound regeneration in deep burns & chronic wounds	No (Semi)	[6, 11, 164]
	TissueMend™	Collagen	Allogeneic			Tissue remodeling for tendon and ligament repair	Yes	[128, 129]

**Integra** ^**®**^ is an artificial skin dermal replacement composed of non-living ECM of porous bovine Type I collagen and a glycosaminoglycan (chondroitin-6-sulfate) with a disposable silicone epidermal layer [7, 124, 148, 149]. The dermal collagen matrix is incorporated and becomes vascularized while the silicone epidermal membrane is temporary and subsequently replaced by a thin layer of autograft [124, 148, 150]. Integra^®^ is used for coverage of burn wounds particularly those requiring partial and full thickness repairs, and also successfully for chronic ulcer treatment of diabetic foot ulcer (DFU) [124, 148]. Integra presents low risks of immunogenic responses and no immunologically significant increase of antibody activity [150].

**Alloderm** ^**®**^ is an artificial skin of acellular collagen matrix containing an entire basement membrane complex used as a scaffold for dermal tissue remodeling [124, 148]. Alloderm^®^ is composed of human allograft skin tissue processed from fresh cadaver skin to remove the epidermis cellular material and freeze-dried after processing [124, 148]. It is used for resurfacing of full thickness burn wounds and temporary wound covers [124]. Alloderm^®^ is accepted by the recipient’s immune system since the allogeneic cells, and antigenic materials are removed rendering it immunologically inert, and therefore reducing the antigenic response [123, 124, 148].

**GraftJacket** ^**®**^ and **GammaGraft™** are artificial skins composed of cryopreserved human cadaveric dermal collagen matrix similar to Alloderm^®^ and are used for various wounds as temporary skin grafts [123]. **Apligraf** ^**®**^ is a culture-derived human bi-layered skin equivalent containing keratinocytes on the upper epidermal layer, fibroblasts on the bottom dermal layer and a matrix of bovine Type I collagen [148, 151]. Apligraf^®^ is employed as an epidermal substitute in the treatment of partial to full thickness burns, chronic wounds, venous leg ulcers as well as diabetic foot ulcers [7, 124, 148]. It promotes healing by providing in addition to ECM components, essential growth factors, and cytokines including TGFα/TGFβ, IL-1, IL-6, and IL-8 [151]. Since Apligraf^®^ does not contain any APCs, it does not cause immunological rejection or support any significant humoral or cellular immune responses [151, 152]. Apligraf^®^ is considered immunologically inert as it does not contain APCs, and thus not cause immunological rejection or support induction of significant humoral or cellular immune responses [151, 152]. Studies have shown the absence of humoral or cellular responses to keratinocytes or fibroblasts of Apligraf^®^; however, safe and reliable human cell sourcing represents a well-recognized problem [153, 154]. Moreover, in clinical trials performed using Apligraf^®^, no signs or symptoms of rejection were detected in vitro to bovine collagen or alloantigens expressed on keratinocytes or fibroblasts in Apligraf^®^ [155, 156]. However, Apligraf^®^ requires joined grafting with an autologous epithelial supplier because the grafted allogeneic cells are temporary [12, 157].

**OrCel™** is a bi-layered skin construct where the dermis is composed of cultured neonatal keratinocytes and fibroblasts derived from foreskin tissues that are seeded into a Type I collagen matrix [7, 120]. OrCel™ is used for reconstruction in recessive dystrophic epidermolysis bullosa and healing of autografts donor sites [120]. Similar to Apligraf^®^, OrCel™ promotes healing by mimicking cytokine and growth factor expression in the healing skin (TGF-α, fibroblast growth factor-1, keratinocyte growth factor-1, etc.) [12, 124]. **PriMatrix** ^**®**^, **Oasis** ^**®**^, and **TissueMend™** are other available collagen matrix-based skin substitutes, which also do not exhibit immunological rejection. PriMatrix^®^ is a fetal bovine dermal substitute comprising extracellular Type I and III collagen matrix scaffolds and used for the coverage of complex wounds to stimulate vascularization and dermal regeneration [122, 158, 159]. Oasis^®^ is an acellular dermal substitute fabricated from porcine small intestine [7, 122, 148]. It is composed of a matrix of collagen (Types I, III and V) and growth factors (TGF-β and fibroblast growth factor-2) processed to remove cell components [122, 148] and commonly employed for wound covering in lower limb wound treatment. Oasis^®^ is decellularized and therefore does not elicit immunological responses. TissueMend™ is an acellular skin substitute with collagen matrix scaffold used for tendon and ligament repair tissue remodeling [160, 161]. Because TissueMend™ is depleted of all cellular components and immunogens, it does not elicit inflammation and foreign body reactions [161].

#### Cross-linked and complexed collagen

Due to the fast biodegradation rate of untreated collagen scaffolds often accompanied by the loss of mechanical strength, various modification techniques have been used to enhance its biodegrading rate, optimize its mechanical property and increase its cellular integration. These include cross-linking treatments or the combination of collagen with other natural or synthetic polymers [143, 162, 163]. Various cross-linking methods have been explored including, but not limited to, collagen scaffolds cross-linked with glutaraldehyde (GA), (1-ethyl-3-(3-dimethylaminopropyl) carbodiimide (EDC) alone or with N-hydroxysuccinimide (NHS), or electrospinning [163–166]. These scaffolds are fabricated with collagen or a mixture of collagen and polymer (chitosan, PLGA, PEG) [167–171], elastin protein [166, 172] or other ECM constituents (hyaluronic acid, glycosaminoglycans) [169, 173–175] and are freeze-dried and treated with GA, EDC/NHS or electrospun for cross-linking. Cross-linked scaffolds decrease biodegradation and increase biocompatibility [162, 164]. Moreover, they efficiently accelerate cell infiltration and proliferation and decrease the inflammatory reaction [167, 168, 173]. Biobrane^®^, TransCyte^®^, EZ Derm^®^, Permacol^®^, and Matriderm^®^ are some commercially available skin substitute employing cross-linked or complexed collagen matrix (Table 1).

**Biobrane** ^**®**^ is a dermal biosynthetic skin substitute which contains Type I porcine collagen packing an inner dermal layer of a 3D nylon filament that is also partially imbedded in an outer epidermal layer of an ultrathin silicone film [7, 124, 176]. Biobrane^®^ is used for partial and full thickness burn wound dressing, particularly in the pediatric population as well as for chronic ulcers for which it provides temporary wound repair and regeneration [7]. **TransCyte** ^**®**^ is a temporary skin substitute made of a synthetic polymeric epidermal membrane and human neonatal fibroblasts cultured on a scaffold of porcine collagen coated with bio-absorbable polyglactin and containing a silicone covered nylon mesh attached to it [12, 149, 176]. Within the nylon mesh, fibroblasts proliferate, secrete matrix proteins/growth factors and are inactivated by freezing before grafting [148, 176]. TransCyte^®^ is used for temporary wound dressing of partial thickness burns [124, 148, 176]. **EZ Derm** ^**®**^ is an acellular xenogeneic (porcine) dermal matrix composed of an aldehyde cross-linked collagen matrix [12, 148, 177] used for the temporary dressing of partial thickness burns [177]. Porcine products do not undergo vascularization, and the aldehyde cross-linking treatment allows prevention of host immune responses and, consequently, no rejection [177]. **Matriderm** ^**®**^ is composed of an extracellular bovine Type I collagen matrix with elastin and used for full thickness burn and chronic wounds [7, 122]. **Permacol** ^**®**^ is an acellular porcine-derived dermis with collagen and elastin matrix used as a temporary skin dressing [122, 149].

#### Fibronectin and fibrin

Fibronectin is a ubiquitous glycoprotein and a major multifunctional constituent of the ECM [144, 178]. Fibronectin has multiple functions including, in particular, promoting the adhesion, proliferation, and contraction of cells (macrophages, fibroblasts, etc.) that participate in wound healing [178–180]. Moreover, fibronectin interacts with several growth factors and therefore regulate their expression and serve as reservoir increasing their local bioavailability [181]. However, since fibronectin is inhibited by mechanical stretching and fails to promote vascularization, there is a limited number of fibronectin-based biomaterials available [121, 144]. Nevertheless, we can include the use of fibronectin associated with fibrin as a matrix to support skin cell growth (keratinocytes and fibroblast) for skin replacement [121, 182]. Fibrin, a fibrous protein derived from soluble plasma fibrinogen, which supports keratinocytes and fibroblast proliferation and migration in wound healing is also a potential source of natural biomaterials for skin substitute [144, 183]. The fact that fibrin is autologous and a potent source of growth factors required for wound healing is a net advantage for using a fibrin matrix [144].

**MyDerm™** is a fully autologous bi-layered living engineered skin substitute employing fibrin as the scaffold [144, 184]. It is constructed using keratinocytes, and fibroblasts skin biopsied cells and fibrin from the patient’s plasma as biomaterials [144, 184, 185]. MyDerm™ is suitable for coverage of full-thickness skin loss [144, 184] and is assimilated and integrated into the patient’s skin without causing immune rejection and cross-contamination [144, 184]. Fibrin is also used as a sealant in tissue-engineered skin substitutes [182, 186] to mimic the final coagulation cascade step where soluble fibrinogen is converted into insoluble fibrin by thrombin [186, 187]. The sealant polymerizes in a semi-rigid fibrin clot, thus serving as a sealing barrier to prevent leakage from the skin structure [186, 187]. Fibrin sealant presents significant advantages including biocompatibility and biodegradability, without inducing inflammation and foreign body reactions [187]. Fibrin sealant is used with skin grafts to improve the fixation and uptake of the graft and in tissue engineering of skin, substitutes to accelerate wound healing [182, 188, 189]. **BioSeed** ^**®**^ is an autologous skin substitute consisting of cultured autologous keratinocytes re-suspended in a fibrin sealant and is mainly used for wound treatment, e.g., chronic leg ulcers [190, 191].

#### Hyaluronic acid (HA)

HA is a ubiquitous linear polysaccharide composed of repeating β-1, 4-linked D-glucuronic acid (GlcA) and β-1, 3-linked N-acetyl-D-glucosamine (GlcNAc) disaccharide units and constitute a part of the ECM [192–195]. HA has several vital functions in the organization and maintenance of the structural integrity of the ECM via interactions with matrix components and skin cells [195]. They include, in particular, the maintenance of tissue homeostasis and hydration as well as binding to cell surface receptors, interleukins and growth factors to activate various signaling pathways that mediate amongst others tissue development, inflammation and wound healing [192, 194–196]. Moreover, HA offers many advantages, notably, including biocompatibility, biodegradability, and susceptibility to chemical modification and cross-linking, which have resulted in HA-based biomaterial scaffolds and skin tissue bio-constructs exhibiting rare adverse effects and antigenic reactions [194, 195]. Some HA-derived materials are thus commercially available mostly for skin replacement in wound healing (Table 1).

**Laserskin** ^**®**^ is a thin and transparent epidermal substitute sheet of benzyl esterified HA derivative [193, 197] whose surface area is cultured with autologous keratinocytes and/or allogeneic fibroblasts and applied to the wound in an inverted fashion [193, 197]. Laserskin^®^ is successfully used for dermal regeneration and skin resurfacing to treat burn wounds or chronic full-thickness ulcers without adverse effects and antigenic reaction [193, 197–199]. **Hyalomatrix** ^**®**^ is a bi-layered acellular dermal substitute of the hyaluronan-based scaffold with a temporary external layer of silicone, which acts as an epidermal barrier [7]. Hyaluronan-based scaffold incorporates into the wound, delivers hyaluronan and induces the formation of neodermis [196]. Hyalomatrix^®^ is clinically used to stimulate the healing process in deep burns and chronic wounds treatments [7, 200]. **Hyalograft 3D** is also an acellular dermal skin substitute composed of a bilayer of hyaluronan-based scaffold [105]. Unlike Hyalomatrix^®^, it lacks the pseudo-epidermal silicone layer but has autologous fibroblasts that secrete the necessary growth factors/cytokines to sustain the healing wound [201]. Hyalograft 3D is used mainly in conjunction with Laserskin^®^ for deep burns and foot ulcers treatment [105, 196]. Both Hyalograft 3D and Hyalomatrix^®^ are biocompatible and biodegradable and do not induce any foreign body reactions since their components are acellular [12].

#### Skin substitutes with synthetic biomaterials

The use of polymers to fabricate hydrogels scaffolds is another promising alternative in skin tissue engineering. Hydrogels matrix scaffolds have been developed and exhibit greater properties including 3D network structure with structural resemblance to ECM, high permeability and diffusion for oxygen and nutrients, precise design and control of mechanical properties, and excellent biocompatibility and biodegradation [13, 127, 129, 202]. Biomaterials used for making hydrogel scaffolds range from synthetic polymers including PEG, PLGA and natural polymers like dextran, chitosan, gelatin, fibrin, and hyaluronic acid [127–130]. Natural polymers have unique advantages because they are biocompatible, biodegradable and have crucial biological functions. Nonetheless, their use is limited by their potential immunogenic reactions and relative inability to form mechanically stable constructs [127, 203]. In contrast, synthetic polymers possess superior mechanical properties but often lack natural ligands and/or biological epitopes to interact with cell receptors or soluble proteins [203, 204]. Consequently, a combination of natural and synthetic hydrogels is often used, thus producing a cellular responsive hydrogel matrix exhibiting excellent mechanical and structural properties with high biocompatibility and bio-functionality [204, 205].

Porous and nanometer-sized fibrous matrix scaffolds have been fabricated to support skin tissue formation for skin wound repair and more importantly for slow release of essential growth factors required for tissue regeneration [206]. Nanofibrous scaffolds are made of natural and synthetic polymer complexes: poly(L-lactic acid)-co-poly(ε-caprolactone) (PLA-PCL) and gelatin; collagen and chitosan; PCL and collagen; PCL and PEG; PCL and collagen; chitosan and polyvinylalcohol (PVA); PEG and fibrinogen and others [203, 206, 207]. Furthermore, nanofibrous scaffolds contain open controllable cellular pores allowing an endowed cell adhesion and proliferation to form new tissues [206]. The scaffold material is then seeded with skin cells (keratinocytes/fibroblasts) and/or functionalized with growth factors or cytokines for their controlled delivery [206]. The scaffold undergoes degradation and absorption [206], and although most of them increase the inflammatory response [206, 208], no immunogenic reactions or rejection have been reported.

Other forms of hydrogel scaffolds that have developed and tested include bi-layered scaffolds composed of chitosan for reconstructing severe burns which exhibited a reasonable tolerance of chitosan and tissue regeneration [209]. Moreover, several skin substitutes using polymer-based scaffolds are currently available in clinical practices (Table 1). Examples include Dermagraft^®^, PolyActive and MySkin™. **Dermagraft** ^**®**^ is a cryopreserved skin substitute made with living cultured human neonatal fibroblasts and plated onto a bio-resorbable polyglactin mesh scaffold [151, 210]. Dermagraft^®^ does not present any evidence of rejection or adverse reactions and is used for burn and chronic wounds and chronic diabetic foot ulcers [7, 151, 210]. **PolyActive** is a bilaminar skin substitute made of autologous cultured keratinocytes and fibroblasts seeded into a porous matrix of polyethylene oxide terephthalate and polybutylene terephthalate components [211]. PolyActive is used for partial-thickness wound dressing and uses autologous cells and biodegradable synthetic dermal components, therefore it does not pose potential risks of immune rejection [12]. **MySkin™** is an autologous skin substitute consisting of autologous human keratinocytes cultures seeded on a silicone polymer support layer and is used for neuropathic, pressure and diabetic foot ulcers [212].

#### Stem cells in the development of perfect skin and avoidance of immune rejection

Skin regeneration growth and repair are evolutionary processes, but scarring is an ultimate consequence. In cases of severe skin injuries, a large portion of the skin is damaged, thus rendering it prone to infections and devoid of performing its basic thermoregulation function. Current options available for severely damaged skin replacements are autologous grafts or allogenic skin grafts where recipients are treated with immune-suppressants to prolong survival of transplant. Nevertheless, immunosuppressant treatments are toxic to skin recipients with chronic disabling diseases leading to infections and cancer [213, 214]**.** To avoid immune rejection, tissue biologists now employ cadaver skin therapy capable of surfacing full thickness burns known as **Alloderm** ^**®**^ (as discussed above). Other commercially available skin options used to avoid immune rejection are **Permacol** ^**®**^, which is a porcine-derived acellular matrix, and **Apligraf** ^**®**^ (organogenesis) human allogeneic neonatal foreskin fibroblast [121]. Despite these research endeavors, these skin substitutes are not fully capable of solving the problems of graft rejection. Tissue engineering of artificial skin to mimic natural skin and which is immuno-compatible is emerging as the solution for skin graft rejection [215]. However, challenges are still eminent in designing tissue-engineered donor skins to match the recipient HLA gene complex system, which codes the MHC complex of human responsible for regulation of immune system, or making modifications in the genetic makeup so that there are neutral surface receptors.

Advancement in tissue engineering and cell biology after three decades has resulted in many alternatives to wound healing and tissue regeneration. Ideally, skin replacement should functionally and physically mimic natural skin, be resistant to infection, have vascularization, hair follicles, sebaceous glands, and more importantly lack of antigenicity [216]. Skin replacements that are commercially available can protect the wound and help to reestablish epidermal and dermal layers, but they lack sweat glands, blood vasculatures, and hair follicles.

The emergence of stem cells with transformation capacities into different tissues and organ systems of the body, make them exceptionally attractive for human biomedical applications, including skin regeneration. Development in cell biology has made mesenchymal and embryonic stem cells technologies bring some surety to complete skin regeneration, mainly by increasing the chances of developing autologous skin grafts with reduced chances of immune rejection [217].

#### Mesenchymal stem cells (MSCs)

The seminal findings from a study conducted by the 1960 Nobel laureate, Peter Medawar paved the way for modern organ and tissue transplantation [143]. In that study, a recipient of allogeneic skin graft transfused with bone marrow from a skin graft donor resulted in the induction of immune tolerance by generating possible chimeric immune cells and thus the avoidance of immune rejection. MSCs are components of the bone marrow known for their immune tolerant or hypo- immunogenic or immune-privileged properties. These properties of MSCs potentially can be exploited for graft transplantation to avoid MHC barriers and creation of off-the-self artificially constructed skin. Recent studies show that the hypo-immunogenic property of MSCs does not prevent immune rejection but instead delay the process. Still there are clear advantages of using autologous MSCs and differentiating them to become perfect skin [218]. MSCs are also advantageous over fibroblast and other cell types in regenerative medicine because they can direct immune responses to suppress maturation of DCs, T and B lymphocytes, and NK cells [219]. Ryan and colleagues [220] reported that the hypo-immunogenic property of MSCs is due to three attributes in that they 1) often lack MHC-II and co-stimulatory molecules expression, 2) prevent T cells activation by indirect modulation of DCs and NK cells and, 3) generate production of the immunosuppressive cytokine IL-10, prostaglandins, and indoleamine 2,3,-dioxygenase, which reduced the availability of tryptophan in the local niche ultimately giving MSCs the potent armory to avoid allogenic immune responses. These MSCs attributes nonetheless, exhibit some degree of immune tolerance in allogeneic grafts and will require additional evaluations before being used in clinical studies. In mouse skin graft models, allogeneic skin grafts not treated with the immunosuppressant, cyclosporine showed immune rejection with elevated levels of IFN-γ and IL-2 [221]. Results from a comparative study by Chen et al., [222] using allogenic and syngeneic bone marrow-derived MSCs and fibroblasts showed that MSCs isolated from autologous and allogeneic mouse models enhanced wound repair and regeneration. The levels of infiltrated CD45+, CD3+ and CD8+ T cells were comparable in cases of bone marrow-derived MSCs but significantly increased in allogeneic transplanted fibroblasts, suggesting lack of immune response to MSCs. Although MSCs are important in wound healing and repair, their hypo-immunogenic characteristic is dependent upon the specific route of administration for tissue/organ regeneration. Majority of the above mentioned studies have concluded that MSCs are immunotolerant in the initial stages or primary immune response as documented from both in vivo and in vitro experiments [219–221]. But, limited MSCs studies have addressed investigating the secondary immune response. For example, normal and diabetic mouse models administered MSCs via the tail vein or the pancreatic routes exerted low immunogenicity and immunosuppressive properties during the initial period of transfusion [223]. However, during the later stage, mice receiving MSCs via the pancreatic route produced insulin and expressed MHC II, generating significant T cell responses. In contrast, mice receiving MSCs by tail vein remained immune-privileged. These results underscored how differences in the transplantation routes and microenvironments can influence the immunogenicity of MSCs, thus making them attractive for artificial skin regeneration. MSCs reportedly prolonged skin grafts survival time also in a nonhuman primate baboon model [224]. MSCs and regulatory T cells function collectively to drive the immune system thereby increasing the probabilities for allograft survival [225].

Umbilical cord Wharton’s Jelly, an excellent source of stem cells (WJ-MSCs), isolated by removal of both arteries and vein [226] have been shown to exhibit similar properties as those described above for MSCs. In addition, WJ-MSCs can differentiate into cells of mesoderm, ectoderm and endoderm origins [227]. Stem cells isolated from WJ-MSCs are well tolerated by Severe combined immunodeficiency **(**SCID) mouse and they do not induce adverse reaction after transplantation and in vitro soft agar assays [227]. Moreover, WJ-MSCs treated with inflammatory cytokines exhibited higher activity of immunomodulation as compared to treated bone marrow-derived MSCs. Growing WJ-MSCs on de-cellularized amniotic biological scaffold induced scar free wound healing, hairs and better biomechanical strength after transplantation onto SCID mouse than did MSCs alone [226]. Other MSCs such as adipose tissue-derived stem cells (ADSCs) were revealed to be immunosuppressive*,* thereby making their use appealing for transplantation without employing cytotoxic drugs [228]. Prior treatment of animals with a single dose of ADSCs before skin transplantation prolonged their skin transplants survival by expansion of CD4+ Tregs, IL-10 production and suppression of Th17 responses [228]. Overall, MSCs are attractive for regeneration of perfect dermal replacement and have been tested in commercial artificial skin substitutes [229–231].

#### Embryonic stem cells (ESCs)

ESCs developed from the inner cell mass of mouse blastocysts were described in 1981 [232] followed by the first derived human ESCs (hESCs) in 1998 [79]. However, there are lots of ethical questions associated with using human fetus for regeneration of artificial organs. It is also difficult to generate tailored-specific ESCs for treatment of specific diseases or patients. We can address this issue by inducing pluripotency in adult stem cells by direct remodeling. Somatic cells can be remodeled to an embryonic-like status by transfer of nucleus from somatic stem cells to oocyte. [233–235] or by fusion with ESCs [236]. Researchers cloned mice by injecting nuclei from hair follicle and keratinocytes and showed that skin somatic stem cells can easily differentiate into whole organisms [237]. In addition, stem cells nuclei can be redesigned to pluripotency by exposing them to unfertilized oocytes cytoplasm as discussed later in the review. ESCs, with its self-renewal and pluripotent capabilities, are an encouragement for tissues/organs regeneration and their ability to differentiate into a variety of cell lineages has stimulated research in generating neurons [238], cardiomyocytes [239], hepatocytes [240], hematopoietic progenitor cells [241] and skins [242, 243].

ESCs are believed to be immune privileged cells albeit with conflicting results. Experiments using undifferentiated and differentiated cells in a mixed lymphocyte reaction (MLR) showed limited or absence of human peripheral blood mononuclear cells (hPBMCs) and human peripheral blood lymphocytes (hPBLs) proliferative responses, which were attributed to diminished MHC class II expression levels by hESCs [241]. In opposite to this, MLR performed with added CD4+ T cells and DCs mixed with hESCs demonstrated not only that hESCs lacked inhibition of T cells proliferation, but they also induced their proliferation [244]. This may be because hESCs express MHC class I, but do not express MHC class II and costimulatory molecules; whereas mature DCs display both MHC class I and II, and costimulatory molecules such as CD80, CD86, and CD40, which confer upon them the potent capacity for T-cell activation.

The pluripotent capability of ESCs highlights their potential applicability for future therapeutics in tissue regeneration to treat numerous severe illnesses. Similarly, the immunogenicity of ESCs represents one of the major obstacles precluding the successful translation of ESCs-based therapies. The immunogenic characteristics of ESCs are dynamic and in constant flux depending on their differentiation state and the environment surrounding them. When ESCs are undifferentiated, their high proliferation rate and low expression of potentially immunogenic surface proteins present an elusive target for the immune system. However, after differentiating and immunogenic cell surface markers are increased, ESCs are at increased risk of immunologic rejection. hESCs can be best used for regenerative medicine therapy as suggested by Taylor et al. [245] by creating hESCs bank typed with human leukocytes antigen to avoid immune rejection.

#### Induced pluripotent stem cells (iPSCs) to escape immune rejection

**Fig. 3:**
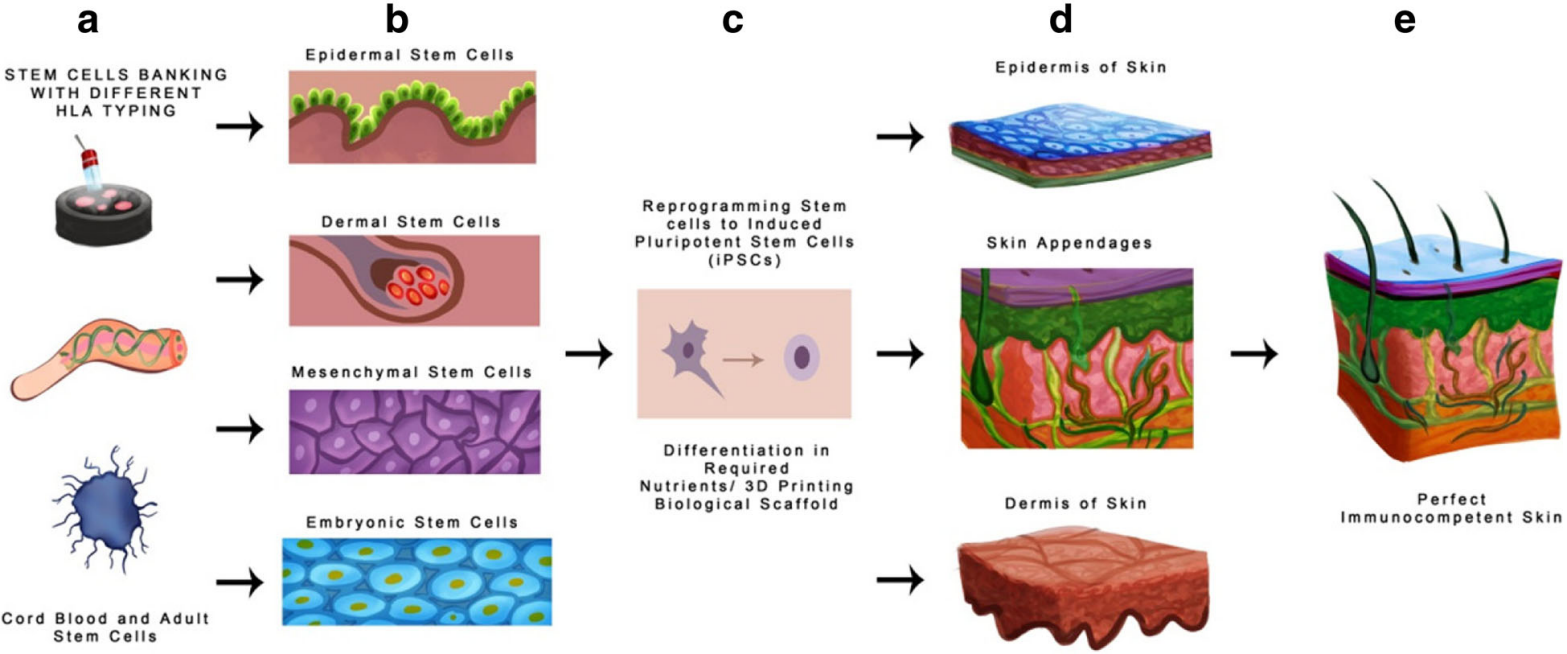
Strategies to develop immune compatible natural skins substitutes. In the model we discuss the strategy of development of immuno compatible skin by (**a**) Stem cell banking on the basis of human leucocyte antigen of adult stem cells and umbilical cord. **b** Remodeling the stem cells and (**c**) Introducing pluripotency and differentiating cells in to different cell lineage (**d**) Development of three-dimension structure (**e**) Full length lab grown perfect skin

Induced pluripotent stem cells are the most recent development in cell biology wherein remodeling gene expression of somatic cells occurs without modifying DNA into an ESCs stage with multipotent capability. This advancement can resolve ethical and short-coming issues of employing ESCs in regenerative medicine. Vital organs of our body such as brain, skin, bone and skeletal muscles have self-renewal capacity in the form of stem cells, which can regenerate injured tissues and are responsible for normal growth and repair mechanisms [246]. However, their limitations reside in being difficult to culture, lack proliferative capacity, undergo apoptosis after transplantation, inability to develop vascularization and expensive for in vitro maintenance. These limitations prevent their application for artificial skin development and regeneration. Notwithstanding, some of these shortcomings and apprehensions were solved after the discovery of iPSCs in 2006 [247] when Takahashi and Yamanaka introduced four transcription factors (Oct 3/4, Nanog, Lin28, and SOX 2) into mouse fibroblasts resulting in ESCs exhibiting continuous proliferative capacity and differentiated into different cell types. iPSCs have characteristics that are well-suited for regeneration repair since cells from a transplant recipient can be modified by reprogramming them into multi lineages and increasing their chances to reduce immune rejection, which can be further exploited for the treatment of genetic disorders [247, 248]. The continuous modification and progress in iPSCs’ reprogramming modification can give new directions to regeneration, particularly artificial skin implants. The evidence is provided that reveals iPSCs can be differentiated into different cell lineages, which can lead to the formation of fully differentiated 3D skin structures with skin appendages and vascularization [14, 249]. Biology of skin makes it easily accessible, from both patient and normal healthy individual where iPSCs can serve as an ideal platform for regeneration of skin since skin somatic stem cells have an affinity for “Yamanaka factor” than any other system of the body. Somatic stem cells from skin have an affinity towards transcription factors Oct3/4, Sox2, Klf4, and c-Myc that are required for induction of pluripotency in cells with cells other than integumentary origin [249, 250]. Investigators have shown that precursors of melanin along with the hair forming units of our body have high basal expression levels of SOX2 and Klf4 transcription factors, which help to differentiate them easily into iPSCs [251]. Results from another study show that not all ‘Yamanaka factors’ are required for induction of pluripotency into somatic skin cells since this process could equally be accomplished with only the Oct4 factor. Reprogramming of somatic stem cells into iPSCs is also less labor intensive [252]. Studies confirming the possibility of reprogramming somatic cells showed that inducing pluripotency in keratinocytes resulted in regrowth of epidermis after exposure to bone morphogenetic protein 4 and vitamin A metabolite [253, 254]. In a different study, mouse fibroblasts were converted into iPSCs; differentiated into melanocytes and then embryoid bodies when co-cultured with wingless-type 3 and EDN3 stem cell factors [255]. Even though iPSCs show great promise towards organ regeneration and growth, the long-term in vivo compatibility issues are unknown. They display many genetic and epigenetic aberrations that can cause cancerous growth or graft immune rejection. iPSCs are known to induce low levels of immunogenicity, have decreased T cells infiltration and reduced expression of JCLN1 and NOHA genes that are responsible for immunogenicity, and suppressing skin and teratoma tissues [233]. Qiau Lu et al., [256] reported generation of hypo-immunogenic hiPSCs by exposing them to allogenic hPBMCs. These cells expressed reduced MHC class II, IFN-γ, TNF-α, and IL-17; moderate MHC class I and HLA-G co-stimulatory molecules and high levels of IL-10 from Tregs in comparison to human skin fibroblast. So far, we have made significant advancements in developing strategies for culturing and reconstruction of 3D skin biological constructs that bear similarities to the normal competent skin (Fig. 3). Additionally, we are now using somatic stem cells to develop dermal and epidermal compartments of skin to treat burn patients [257].

## Conclusion

Skin graft rejection remains an important challenge in regenerative medicine. The rejection process occurs by activation of T cells by way of the direct, indirect or semi-direct alloantigen recognition pathways, as well as the active participation of accessory B and NK cells that destroy donor cells. Several attempts of inducing tolerance and prolonged survival of skin transplants have been made, such as therapies addressing donor-derived DCs and the inactivation or deletion of their reactive T cells. Such strategies have led to remarkable progress in the understanding and control of skin grafts rejection. Nonetheless, despite the progress made on the induction of long-term allografts survival, they have not provided robust tolerance and the skin graft survival achieved is not indefinite. Other alternative strategies have been more successful such as stem cell-based therapies that allow functional repair of skin after severe burn injury. Stem cells therapy holds great promise for bioengineered skin, because of ease of availability for the repair and replacement of damaged skin. Most cellular and acellular skin substitutes currently available on the market help to repair damaged skin by providing protection from infection and aiding in wound healing. Nevertheless, they are unable to provide complete skin functionality as well as sensitivity and thermoregulation capacities. With the discovery of iPSCs, stem cells banking could potentially resolve the issues of graft rejection and provide a viable option for autografts. Alternatively, there is now a significant number of bioengineered skin substitutes used clinically for skin repair or skin replacement therapies. To achieve a definitive regeneration of skin, however, still requires combining two or more procedures. Despite multiple advantages offered with bioengineered skin substitutes, there is no ideally available skin substitute allowing for permanent skin repair that is commercially available. Recent progress, especially in the design of biomaterials for incorporation into skin substitutes coupled with stem cells technology offers hope for more effective approaches in the future.

## Abbreviations

3D: Three dimension
ADSCs: Adipose tissue derived stem cells
APCs: Antigen presenting cells
B-cells: B Lymphocytes
CD4+: Helper T cell
CD8+: Cytotoxic T cell
DCs: Dendritic cells
ECM: Extra cellular matrix
ESCs: Embryonic stem cells
HLA: Human leucocyte antigen
iPSCs: Induced pluripotent stem cells
LCs: Langerhans cells
MHC: Major histocompatibility complex
MSCs: Mesenchymal stem cells
NK: Natural killer cells
SCID: Severe combined immunodeficiency
T cells: T-Lymphocyte
Th1: T helper subset 1
Th17: T helper subset 17
Th2: T helper subset 2
WJ-MSCs: Wharton jelly Mesenchymal stem cells
